# Anti-Inflammatory Effect of Unsaturated Fatty Acids and Ergosta-7,22-dien-3-ol from *Marthasterias glacialis*: Prevention of CHOP-Mediated ER-Stress and NF-κB Activation

**DOI:** 10.1371/journal.pone.0088341

**Published:** 2014-02-13

**Authors:** David M. Pereira, Georgina Correia-da-Silva, Patrícia Valentão, Natércia Teixeira, Paula B. Andrade

**Affiliations:** 1 REQUIMTE/Laboratório de Farmacognosia, Departamento de Química, Faculdade de Farmácia, Universidade do Porto, Porto, Portugal; 2 Laboratório de Bioquímica, Departamento de Ciências Biológicas, Faculdade de Farmácia, Universidade do Porto, Porto, Portugal; 3 IBMC –Instituto de Biologia Molecular e Celular, Universidade do Porto, Porto, Portugal; Cincinnati Children's Hospital Medical Center, United States of America

## Abstract

There has been increasing awareness to the potential interest of drug discovery from marine natural products to treat several pathological conditions, including inflammation. In this work we describe the anti-inflammatory activity of several compounds present in the echinoderm *Marthasterias glacialis* (spiny sea-star), using the inflammatory model RAW 264.7 cells challenged with LPS. Lipidomic profiling of the organism revealed two major classes of compounds: fatty acids and sterols. Among these, the predominant compounds *cis* 11-eicosenoic and *cis* 11,14 eicosadienoic acids and the unsaturated sterol ergosta-7,22-dien-3-ol were evaluated. The mechanism of action of the compounds was distinct as they modulated different levels of the inflammation pathway. Classical inflammatory markers, such as COX-2, iNOS, IL-6 and NF-κB, were evaluated. We also studied the contribution of the CHOP pathway-mediated ER-stress to the inflammatory process. Overall, the sterol ergosta-7,22-dien-3-ol was the most active compound, however maximum activity was obtained when all compounds were tested in combination, thus suggesting a potentially synergistic activity of both classes of metabolites. This work establishes the echinoderm *M. glacialis* as an interesting source of anti-inflammatory molecules.

## Introduction

Inflammation is a complex process occurring in many animals and constitutes one of the first lines of defense against a number of stimuli that are perceived as harmful, such as bacteria, trauma and irritants. While acute inflammatory processes may serve to protect the organism, deregulated or chronic inflammatory processes are the basis of a number of pathological conditions that include asthma, rheumatoid arthritis, cardiovascular diseases, among many others.

In the continuous search of new molecules to counter and treat inflammatory conditions, Nature has been a prolific source of such compounds for many years. Recently, much interest has been given to anti-inflammatory compounds from non-conventional environments, with marine micro and macroorganisms being very important. Several compounds of marine origin have been described in the last few years, such as avarol, cycloamphilectenes 1–6, cavernolide and the lead compound manoalide [1], among many others. Some of these compounds are already in advanced stages of clinical trials.

Several factors are known to modulate the inflammatory process. Among these, NF-κB plays a pivotal role [2], [3]. When free from its cytoplasmic inhibitor proteins, the IκB family, translocates to the nucleus, where it binds to the promoter region of several genes, thus exerting a number of actions. Target genes codify proteins that include cytokines, cyclooxygenase-2 (COX-2), inducible nitric oxide synthase (iNOS), proteases and many others [4].

*Marthasterias glacialis* L., also known as spiny sea-star, is an echinoderm that can be found in several ecosystems. Previous works have addressed the chemical composition of this organism and several classes of compounds important in human diet were found, namely carotenoids [5]–[7], fatty acids, sterols and amino acids [8], [9]. Some biological properties were also evaluated, namely its anticancer activity against both non-human [6] and human cells [8]. In this work the anti-inflammatory effect of a lipophilic extract and its main compounds was evaluated in the LPS-induced RAW 264.7 macrophages model of inflammation. The effect of the extract on several inflammatory markers was assessed.

## Materials and Methods

### Chemicals

Lipopolysaccharide (LPS), acridine orange (AO), Triton X-100, sulfanilamide, dichlorodihydrofluorescein diacetate (DCDHF-DA), 3-(4,5-dimethylthiazolyl- 2)-2,5-diphenyltetrazolium bromide (MTT), palmitic acid (≥99%), *cis* 11-eicosenoic acid (≥99%) and *cis* 11,14-eicosadienoic acid (≥98%) were from Sigma-Aldrich. Ergosta-7,22-dien-3-ol (≥98%) was from BioBioPha Co., Ltd (China). Sodium nitroprusside dehydrate (SNP) from Riedel-de-Haën (Seelze, Germany). *N*-(1-naphtyl)-ethylene-diamine dihydrochloride were obtained from Merck (Darmstadt, Germany). Dulbecco’s Modified Eagle Medium (DMEM), foetal bovine serum (FBS), Dulbecco’s phosphate buffer saline (DPBS), Hank’s balanced salt solution (HBSS) and antibiotic were from GIBCO (Invitrogen, UK). COX-2, iNOS, IKB-α, β-tubulin and CHOP primary antibodies, as well as anti-rabbit secondary antibody, were from Santa Cruz, USA. IL-6 ELISA kit was from AbCam, UK.

### Sample Preparation

*M. glacialis* individuals were collected in west Portugal (Cabo Carvoeiro) in September 2009. Samples were frozen, transported to the laboratory, lyophilized (Labconco 4.5 Freezone apparatus, Kansas City, MO) and powdered using an electric blender. Lyophilized powder (15 g) were extracted with acetone:methanol (7∶3) and the extraction was repeated as many times as necessary to render the powder colourless. Afterwards, the extract was added to a separating funnel with equal amount (20 mL) of an ether:hexane mixture (1∶1). An equivalent volume of 5% NaCl was then added. The mixture was separated into two phases and the aqueous hypophase was collected and re-extracted with the ether:hexane mixture until no further pigment could be extracted. The organic epiphases were then collected and washed with water in order to remove all traces of acetone and evaporated until dryness in a rotary evaporator. All procedures were conducted under low light conditions, at room temperature and the final residue was kept at −80°C in an inert atmosphere (nitrogen).

### Derivatization and GC-MS Analysis

A solution of 1 mg/mL of extract in ethanol was prepared. An aliquot of 50 µL was then transferred to a glass vial, the solvent was evaporated under nitrogen stream and 50 µL of MSTFA were added to the dried residue. The vial was capped, vortexed and heated for 20 minutes at 40°C. All analysis were performed in triplicate. GC-MS conditions were as described previously by our group [9].

### Cell Culture

RAW 264.7 macrophages were maintained in DMEM supplemented with 10% FBS and 1% penicillin/streptomycin and grown in an incubator at 37°C and 5% CO_2_.

For the Wright staining cells were seeded at a density of 3×10^5^ cells/well in 24-well plates. After incubation with different concentrations of the extract at different times, cells were washed twice with PBS. After fixation with cold methanol, at 4°C for 30 minutes, cells were stained with Wright solution. Cells were then mounted with DPX.

### Cell Viability

Cells were cultured in 96-well plates (2×10^4^ cells/well) and allowed to attach for 24 hours. After this period, cells were treated with LPS, with or without pre-incubation with the extract/compounds for 2 hours. After incubation (24 hours), MTT (0.5 mg/ml final concentration) was added to each well and the plate was incubated for 2 hours at 37°C. The formazan was dissolved by addition of a DMSO:isopropanol mixture (3∶1) and quantified spectrophotometrically at 560 nm. The results of cell viability correspond to the mean of three independent experiments performed in triplicate and are expressed as percentage of the untreated control cells. LDH release was measured after 24 hours in culture media supernatant using CytoTox 96 non-radioactive cytotoxicity assay kit (Promega, Madison, WI, USA) according to the manufacturer’s protocol. Absorbance was read in a multiplate reader at 492 nm. All the results correspond to the mean ± SD of three independent experiments performed in triplicate and expressed as fold increase of absorbance in treatments *versus* untreated control cells, expressed as arbitrary units.

### Acridine Orange

Cells were incubated as described before with LPS, with or without pre-incubation with the extract/pure compounds. After this period, cells were washed with HBSS and incubated with a 0.1 µg/mL solution of acridine orange (AO) for 15 minutes. After this period cells were washed with HBSS and observed in a fluorescence microscope equipped with a 490 nm band-pass blue excitation filter and a 515-nm long pass-barrier filter.

### Nitric Oxide Scavenging Activity and NO Determination

The ability of the extract to scavenge nitric oxide radical generated in a cell-free system was evaluated according to the method previously described [10]. A dilution series (five different concentrations of extract) was prepared in a 96-well plate. The reaction mixtures in the sample wells consisted of 156 µg/mL extract and SNP. The plates were incubated at 25°C for 60 min under light.

Nitrite was quantified by mixing 50 µl of either extract solution or culture media with an equal volume of Griess reagent (1% sulfanilamide and 0.1% naphthylethylenediamine dihydrochloride in 2% H_3_PO_4_) and incubated for 10 minutes in the dark, after which absorbance was read in a multiplate reader set at 540 nm.

### Intracellular Reactive Oxygen Species

Cells were seed in 96-well black plates according to the above mentioned conditions for the MTT assay and exposed to LPS for 24 hours, with or without pre-incubation with *M. glacialis* purified extract for 2 hours. Cells were washed with HBSS 30 minutes before the end of the incubation period, followed by incubation with a solution of DCDHF-DA (25 µM in HBSS) for 30 minutes. Afterwards they were mounted with VectaShield mounting media (Vector, UK). For the quantification of intracellular ROS the plate was read using fluorescence multiplate reader (Excitation: 490 nm excitation; Emission: 520 nm) following incubation with DCDHF-DA. Cells in HBSS were used as negative control and LPS was used as a positive control.

### IL-6

IL-6 levels were determined by ELISA in culture media as *per* the manufacturer’s instructions.

### Western-Blot

Cells were seeded in 6-well plates with a density of 3,5×10^5^ cells/well. Cells were treated for 24 hours with LPS, with or without pre-incubation with extract or pure compounds for 2 hours. After this period, macrophages were washed with PBS, scraped, incubated with a lysis solution with protease inhibitors for 20 minutes on ice and centrifuged at 14,000 *g* for 15 minutes. The supernatant was collected and the protein content determined by the Bradford method. Samples (40 µg) were subjected to 10% SDS-PAGE and proteins were transferred onto nitrocellulose membranes and blocked for one hour at room temperature with a solution of 5% non-fat milk in 0.1% Triton X-100. Overnight incubation at 4°C was performed with antibodies anti-COX-2 (1∶1000), anti-iNOS (1∶100), anti-CHOP (1∶100), anti-IKB-α (1000), anti-tubulin (1∶1000) and then with peroxidase-conjugated secondary antibody (1∶3000) at room temperature for 1 hour. β-Tubulin was used as a loading control. Finally blots were subjected to a chemiluminescence detection kit (Super Signal West Pico; Pierce, Rockford, USA).

### Statistical Analysis

Comparisons of data from different groups, controls and treatments were performed using a one-way ANOVA test. Dunnet’s post-test was used. A *p* value lower than 0.05 was considered statistically significant. All experiments were performed in triplicate with, at least, three independent assays.

## Results

### Extract Concentration Screening and Impact on Cell Morphology

In order to study the anti-inflammatory effect of the extract and its main components, several inflammatory markers were evaluated. Initially, the working concentration of the extract was determined. Given that with the extract concentration of 312 µg/ml a decrease of about 20% of cell viability was noticed (data not shown), in subsequent experiments lower concentrations (156 µg/mL) were used for testing the anti-inflammatory activity. LDH was not present in culture supernatants in any concentration tested (data not shown).

**Figure 1:**
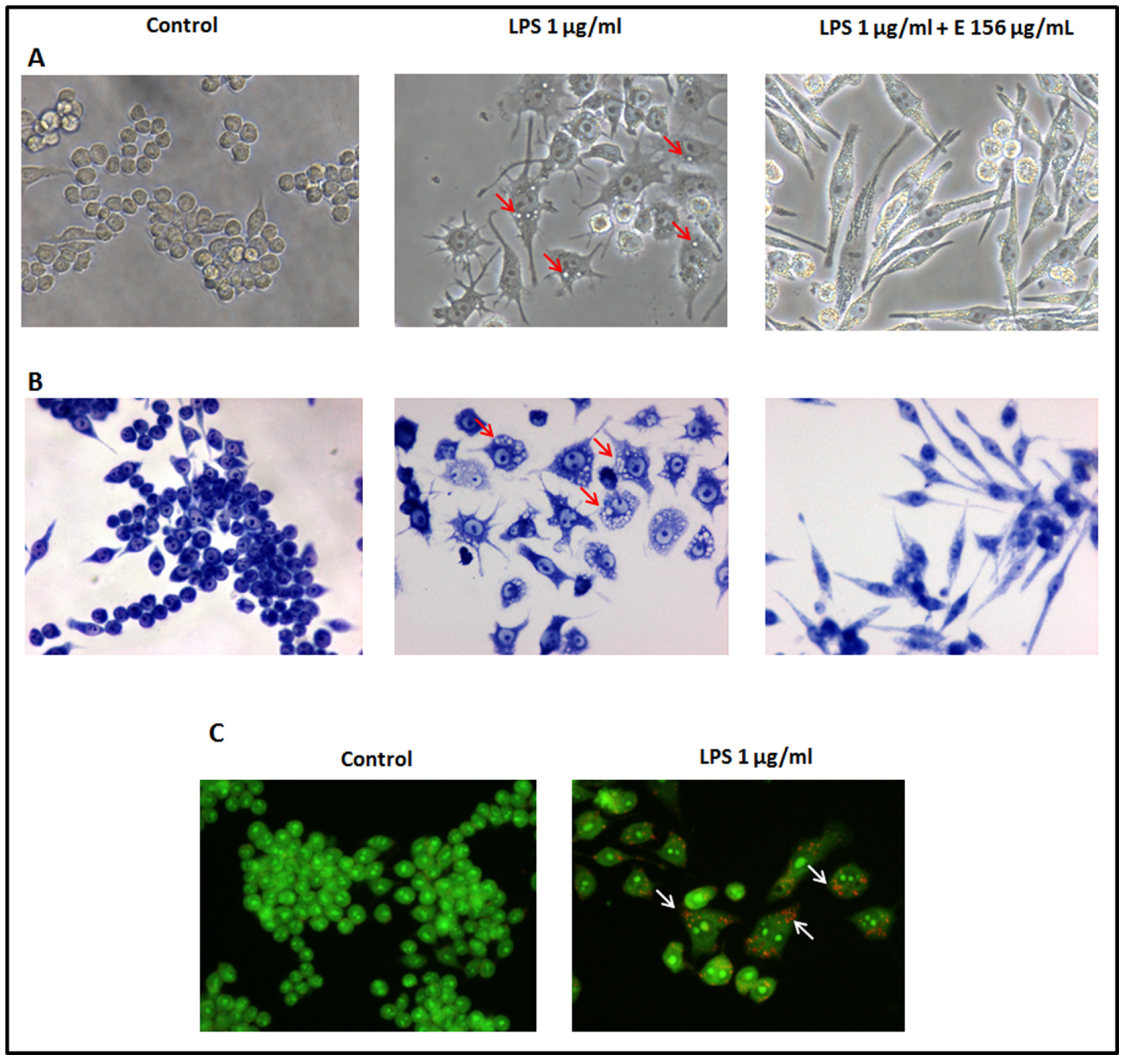
Evaluation of the effect of LPS and LPS+extract on cell morphology: A – phase contrast; B – Wright staining. Red arrows: cytoplasmic vesicles (autophagosomes); C - Acridine orange staining of control and LPS-treated macrophages. White arrows: autophagosomes. Incubation with LPS causes the characteristic morphology of activated macrophages, which includes the advent of autophagosomes. Pre-incubation with the extract attenuates the number of autophagosomes. Original magnification: 400×.

LPS treatment induced alterations in macrophage morphology (Fig. 1), as previously described. Cytoplasmic vesicles were noticed and were compatible with autophagosomes described as a result of toll-like receptor 4 (TLR 4) activation by LPS in macrophages [11]. In order to confirm the identity of these autophagosomes, acridine orange was used to confirm their acidic nature (Fig. 1C). As it can be found in Fig. 1A, pre-incubation of macrophages with the extract caused changes in cell morphology and reduced the number of visible autophagosomes.

**Figure 2:**
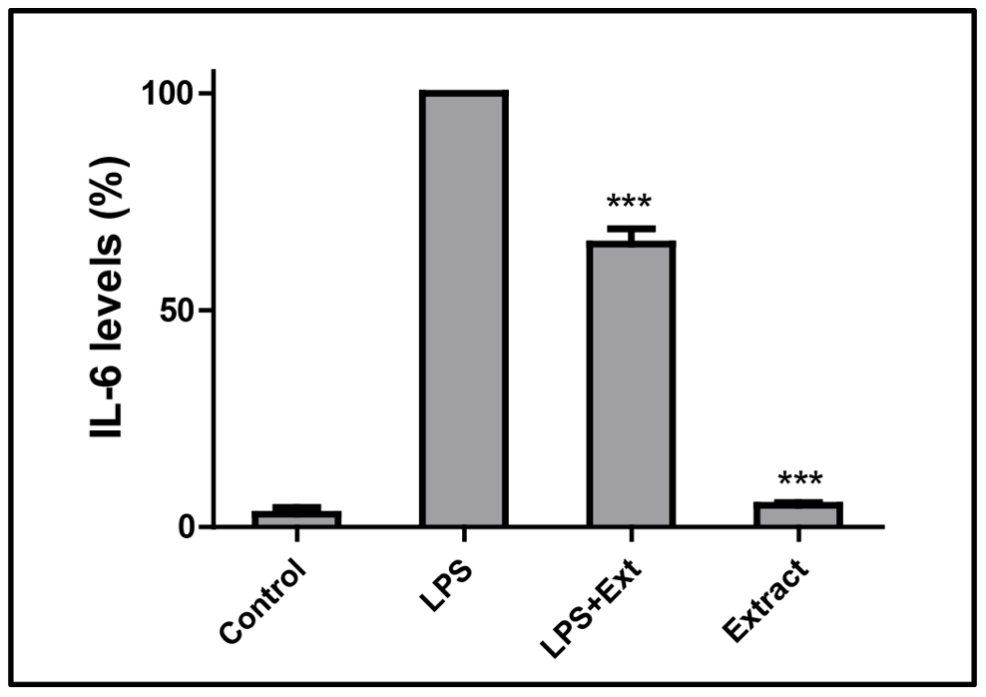
IL-6 levels determined in culture media. Challenge with LPS causes a marked increase in IL-6 levels, which are partly prevented by pre-incubation with the extract (156 µg/mL) for 2 hours. **Ext**: extract (156 µg/mL). The results correspond to the mean ± SD of three independent experiments performed in triplicate. ***P<0.001 (*vs* LPS).

After this point we evaluated the potential anti-inflammatory activity of the extract by screening its ability to prevent/ameliorate LPS-induced increase in IL-6. As it can be seen in Fig. 2, LPS caused a marked increase in IL-6 levels, which were lowered by about 30% in the presence of the extract.

### Effect of LPS and *M. glacialis* Extract/Pure Compounds upon Macrophage Viability

**Figure 3:**
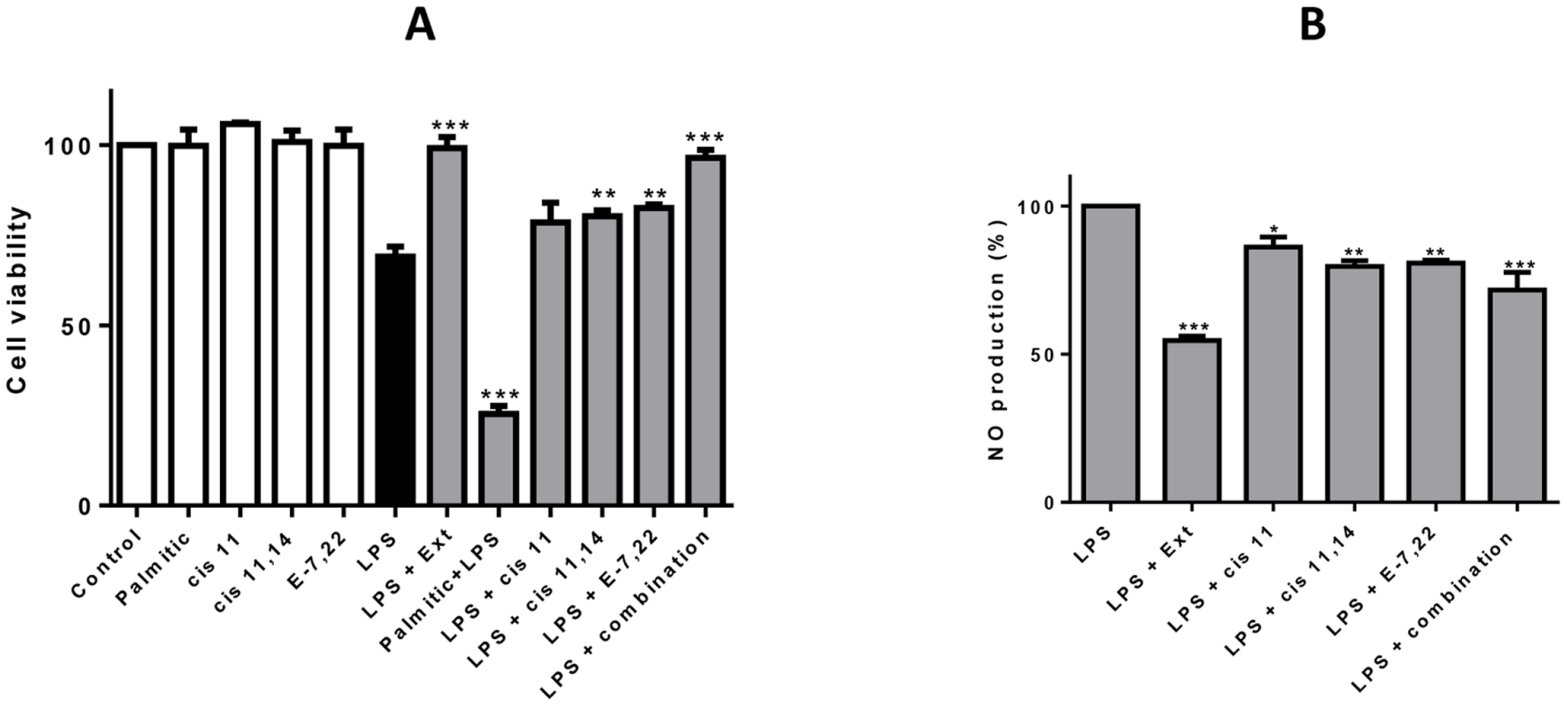
Viability of macrophages after treatment with purified extract or individual compounds with or without LPS after 24 hours of incubation (A). NO production in RAW 264.7 cells in the presence of a purified extract and of the major compounds in the extract, isolated and in combination after 24 hours of incubation (B). Compounds were used in the concentrations at which they occur in the extract; ***cis*** **11**: 35 µM *cis* 11-eicosenoic acid; ***cis*** **11,14**: 10 µM *cis* 11,14-eicosadienoic acid; **E-7,22**: 25 µM ergosta-7,22-dien-3-ol. **Ext**: extract (156 µg/mL); **combination**: Palmitc acid+*cis* 11-eicosenoic acid+*cis* 11,14-eicosadienoic+ergosta-7,22-dien-3-ol.The results correspond to the mean ± SD of three independent experiments performed in triplicate. *P<0.05; **P<0.01; ***P<0.001 (*vs* LPS).

LPS challenge resulted in a decrease of 30% in cell viability when compared to control. This effect was prevented by pre-incubation with the extract at a concentration of 156 µg/mL for 2 hours (Fig. 3A). After this, the individual effect of the main compounds present in the extract was tested. Previous studies have identified and quantified major fatty acids and sterols in *M. glacialis* [9]. We used the same method for the determination of major compounds present in the purified extract used herein. Fatty acids and sterols were major classes in the extract and, at the concentration 156 µg/mL, the major candidates to exert a biological effect were palmitic acid (20 µM), *cis* 11-eicosenoic acid (30 µM), *cis* 11,14-eicosadienoic acid (10 µM) and µM ergosta-7,22-dien-3-ol (25 µM).

All compounds tested individually had no effect in cell viability after 24 hours treatment as assed by the MTT assay (Fig. 3A). When compounds were tested in the presence of LPS, distinct behaviors were found. While *cis* 11-eicosenoic acid had no effect in cell viability, *cis*-11,14- eicosadienoic acid and ergosta-7,22-dien-3-ol had a moderate reduction on LPS-induced loss of viability. Incubation with palmitic acid in a concentration of 20 µM resulted in a marked increase in LPS-induced toxicity. When all compounds were tested together, cell viability was 90% of the control.

### *M. glacialis* Extract/Pure Compounds Modulate NO Levels *via* Down-regulation of iNOS Expression

NO was quantified in culture media after challenge with LPS for 24 hours with or without pre-incubation with the extract for 2 hours. NO was present in control samples in negligible amounts and, for this reason, NO production following LPS incubation was considered 100%.

The extract (156 µg/mL) lowered the values of NO by about 50% of those obtained with LPS (Fig. 3B). After this, we investigated the contribution of the three major compounds: *cis*-11-eicosenoic acid, *cis*-11,14-eicosadienoic acid and ergosta-7,22,dien-3-ol. Palmitic acid was not tested isolated due to its above-mentioned impact in cells (70% loss of viability) although it was included in the mixture of major compounds used to mimic the extract. *cis* 11-Eicosenoic acid only caused a 10% reduction of NO levels, while *cis* 11,14-eicosadienoic acid and ergosta-7,22,dien-3-ol were capable of lowering NO levels by 20%. When all compounds were incubated together, a 30% decrease in LPS-induced NO levels was found (Fig. 3B).

**Figure 4:**
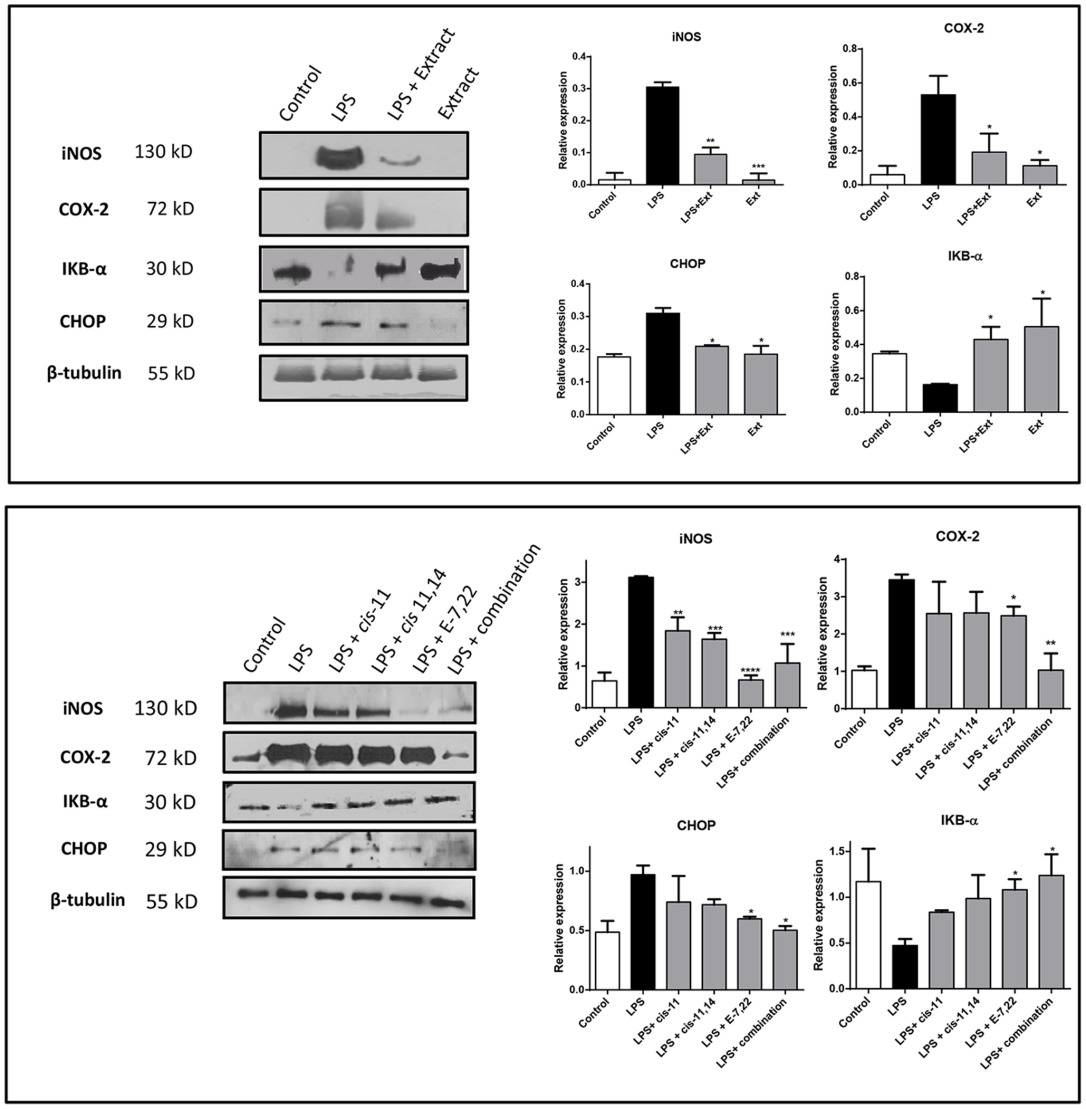
Effect of purified extract (156 µg/mL) from *M. glacialis* (A) and major compounds in the extract (B) in the expression of iNOS, COX-2, CHOP and IKB-α in LPS-treated RAW 264.7 cells. Densitometric analysis of the studied proteins after normalisation to β-tubulin levels. Results are expressed as mean ± SD of three experiments. *P<0.05; **P<0.01; ***P<0.001 (*vs* LPS). **Ext**: extract (156 µg/mL); **PA**: 20 µM palmitic acid; ***cis*** **11**: 35 µM *cis* 11-eicosenoic acid; ***cis*** **11,14**: 10 µM *cis* 11,14-eicosadienoic acid; **E-7,22**: 25 µM ergosta-7,22-dien-3-ol. **combination**: Palmitic acid+*cis* 11-eicosenoic acid+*cis* 11,14-eicosadienoic+ergosta-7,22-dien-3-ol.

Several natural products have displayed NO-scavenging capacity and, for this reason, the NO scavenging activity of the extract was evaluated. NO radical was generated in a cell-free chemical system, as described before [12]. Several concentrations were tested and up to a concentration of 625 µg/mL, far higher than the ranges used in cells, no NO scavenging capacity was found (data not shown). We evaluated the effect of the extract on iNOS levels, the major enzyme responsible for the production of NO. While LPS induced a marked increase on iNOS levels, the extract was able to partially prevent this up-regulation (Fig. 4A). *cis*-11-Eicosenoic and *cis*-11,14-eicosadienoic acids were able to reduce LPS-induced increase in iNOS, albeit in low extent (Fig. 4B). The sterol ergosta-7,22-dien-3-ol had a marked effect in iNOS protein levels, being able to revert those to near-control levels, a behavior also found for the combination of all compounds (Fig. 4B).

### *M. glacialis* Extract/Pure Compounds Down-regulate COX-2

The study of COX-2 levels after LPS elicitation revealed a marked increase in the expression of this enzyme (Fig. 4A), which is in line with the pro-inflammatory effects described for LPS. When LPS was co-incubated with the extract the levels of COX-2 were significantly reduced, a trend that had already been noticed with iNOS protein expression (Fig. 4A). The study of the contribution of the major components of the extract revealed that, although some compounds had a significant activity, notably ergosta-7,22-dien-3-ol, only the combination of all compounds was able to revert the levels of COX-2 to those of control cells (Fig. 4B).

### *M. glacialis* Extract/Pure Compounds Lower LPS-induced ROS

**Figure 5:**
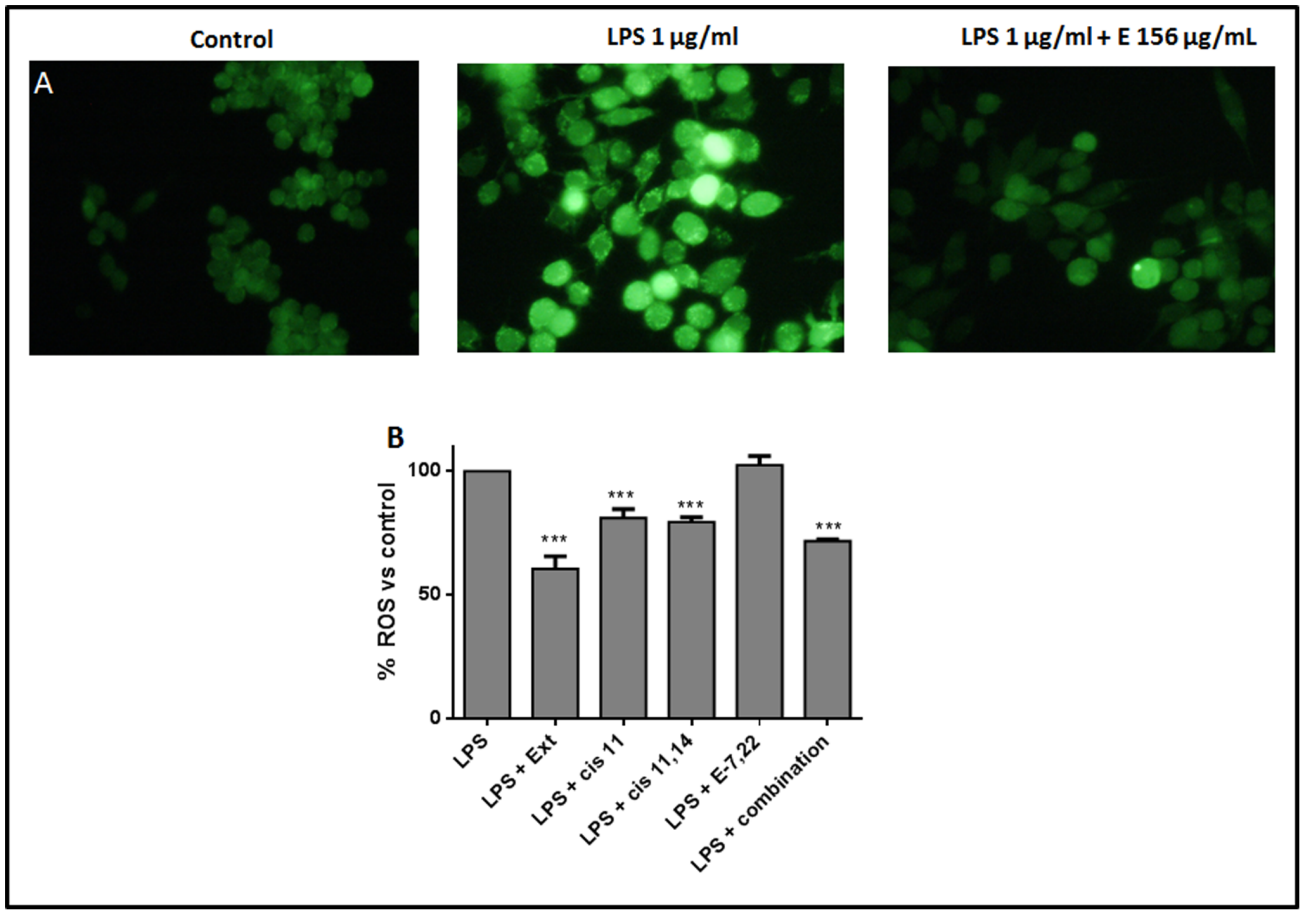
Evaluation of intracellular ROS assessed by the fluorescent probe DCDHF-DA. A – Fluorescence microscopy, qualitative evaluation of the effect of the extract in LPS-activated cells. **B** – Quantitative evaluation of the effect of the extract and its main compounds in LPS-challenged cells. The results correspond to the mean ± SD of three independent experiments performed in triplicate. ***P<0.001 (vs LPS). **Ext**: extract (156 µg/mL); ***cis*** **11**: 35 µM *cis* 11-eicosenoic acid; ***cis*** **11,14**: 10 µM *cis* 11,14-eicosadienoic acid; **E-7,22**: 25 µM ergosta-7,22-dien-3-ol. **combination**: Palmitic acid+*cis* 11-eicosenoic acid+*cis* 11,14-eicosadienoic+ergosta-7,22-dien-3-ol.

We evaluated the effect of both LPS and the extract upon intracellular levels of ROS in RAW 264.7 cells. Initially, cells were incubated with either media, media with LPS or media with LPS after pre-incubation with the extract and cells were then observed in a fluorescence microscope. As it can be seen in Fig. 5A, LPS caused an increase in total intracellular ROS when compared with control cells. Co-incubation of LPS with the extract resulted in a partial reversion of this behavior. These qualitative results were then confirmed by quantification of the signal in a fluorescent multi-plate reader. As shown in Fig. 5B, the extract was capable of preventing LPS-induced generation of ROS by around 50%, an effect that could be mimicked by the combination of all compounds tested. Interestingly, the sterol ergosta-7,22-dien-3-ol had no effect on ROS levels at the incubation time studied herein, with the unsaturated fatty acids causing partial reduction of ROS levels.

### *M. glacialis* Extract/pure Compounds Prevent LPS-induced CHOP Pathway-mediated ER Stress

ER stress has been increasingly implied as a potential source and aggravation of inflammatory processes and CHOP is one of the most commonly used markers of ER-stress [13]. An increase in CHOP expression levels following incubation with LPS was observed, an effect significantly prevented by the extract (Fig. 4A). Unsaturated fatty acids *cis* 11-eicosenoic and *cis* 11,14-eicosadienoic acids were unable to reduce CHOP expression levels in LPS-challenged cells. When cells were incubated in the presence of ergosta-7,22-dien-3-ol CHOP expression was similar to untreated cells (Fig. 4B).

### *M. glacialis* Extract/pure Compounds Prevents NF-κB Activation by Avoiding IκB-α Degradation

**Figure 6:**
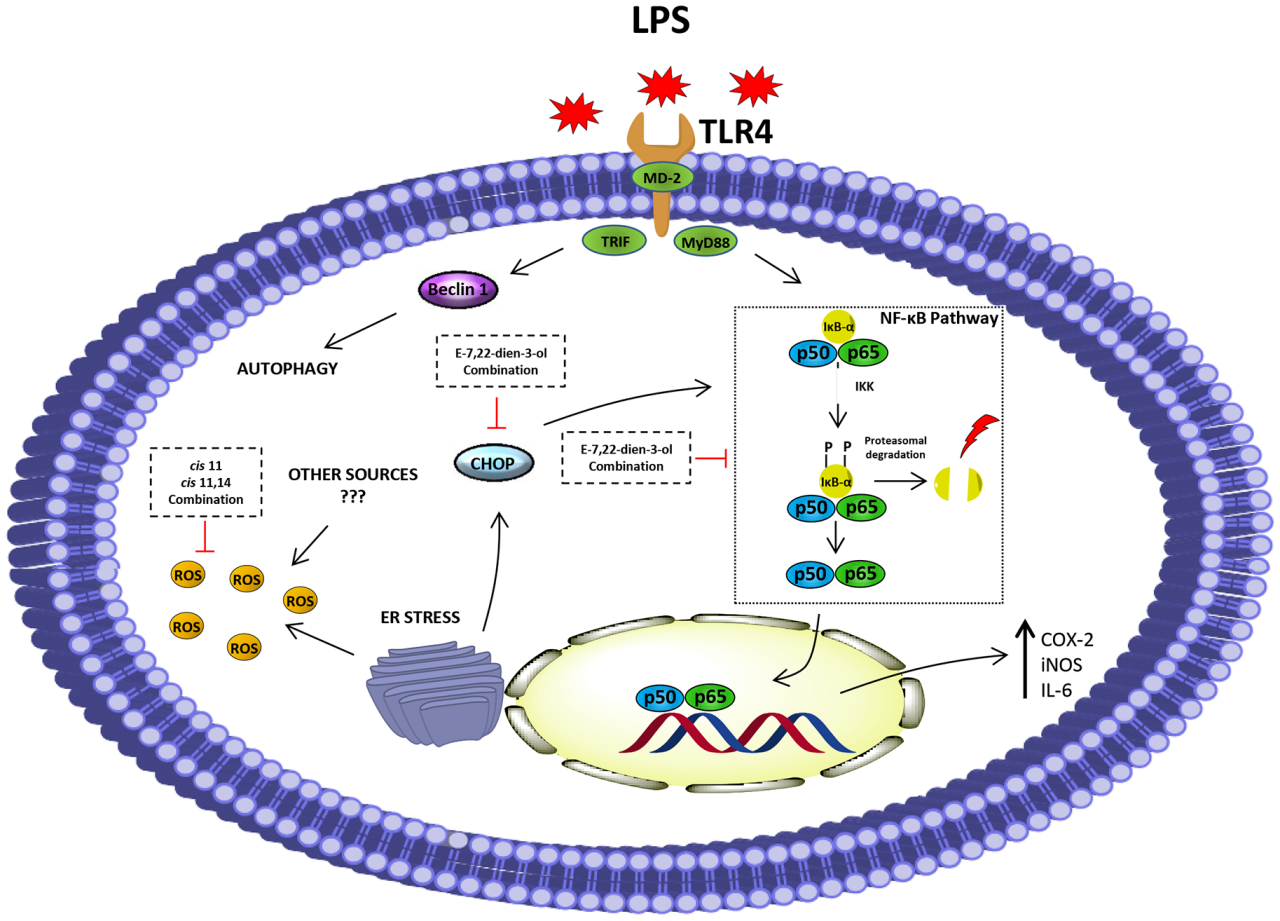
Proposed mechanism for the anti-inflammatory activity of different compounds present in *M. glacialis.*

NF-κB is usually found in its inactive form in cytoplasm by action of the inhibitory protein IκB-α (Fig. 6). As it can be found in Fig. 5, incubation with LPS caused a decrease in the levels of IκB-α, which can explain all the classical traits of inflammatory process, namely the increase of COX-2, iNOS and IL-6 levels. When cells were pre-incubated with the extract, LPS was unable to cause a reduction in IκB-α levels (Fig. 4A). This protective effect was also found when all compounds tested were evaluated in combination (Fig. 4B). These results show that some of the above-presented findings may result from inhibition of the NF-κB pathway.

## Discussion

LPS treatment induced the characteristic morphology of activated macrophages. As a result of TLR4 activation by LPS, the advent of autophagosomes is known to occur. We demonstrated that incubation with the purified extract from *M. glacialis* lowered the number of autophagosomes.

The use of LPS as a pro-inflammatory stimulus resulted in a loss of cell viability of about 30%, which could be prevented by pre-incubation with the extract. When the major compounds of the extract were tested individually, no impact on cell viability was found. Distinctively, in LPS-activated cells different behaviors were found: *cis* 11,14 and ergosta-7,22-dien-3-ol had a protective effect and palmitic acid exacerbated LPS-induced loss of viability. This result suggests a potentially synergistic pro-inflammatory effect of palmitic acid with LPS, which is in line with previous reports that describe palmitic acid as an activator of TLR 4, the same target activated by LPS [14]. When all compounds were combined, reversion of LPS-induced loss of viability occurred, as it had been found for the extract treatment.

Several markers are used for monitoring inflammation, one of them being NO, which is synthesized from the conversion of arginine to citrulline by nitric oxide synthases, the inducible isoform (iNOS) being the main responsible for the large amounts of NO found in inflammatory processes [15], [16]. On the other hand, excessive iNOS activity is known to be related with a number of pathological conditions, such as asthma, psoriasis, neurodegenerative diseases and can lead to NO-induced apoptosis in several cell lines [17]–[19]. We showed that the extract was able to significantly lower the levels of LPS-induced NO as a result of down-regulation of iNOS, an effect for which all compounds tested contributed, notably ergosta-7,22-dien-3-ol.

Other well-known inflammatory marker is COX-2. Apart from COX-2, COX-1 and COX-3 have been described, however in the inflammatory context the inducible isoform, COX-2, is the most significant. We evaluated the effect of both the extract and its compounds in LPS-induced up-regulation of COX-2. As it had been found in the case of iNOS, pre-incubation with the extract prevented LPS-induced increase of the expression levels by over 50%. However, while in the case of iNOS one compound, ergosta-7,22-dien-3ol, was found to be responsible for this activity, in the case of COX-2 no single compound could explain this activity. Instead, the combination of all compounds tested showed higher capacity to prevent LPS-induced up-regulation of COX-2.

In addition, ROS are known to play a pivotal role in early stages of inflammation and in the particular case of LPS, the increase in ROS generation has been shown to be an upstream event taking place following exposure to this positive elicitor [20]. We demonstrated that the purified extract from *M. glacialis* prevented LPS-induced increase in ROS levels by about 50%. Differently from what had been found for other inflammatory markers, ergosta-7,22-dien-3ol had no effect in ROS levels. However, when all compounds were tested together, the activity was comparable to that found for the extract, a trend that had already been found for COX-2 expression levels.

From a cellular point of view, activation of the NF-κB pathway is one of the most important events that trigger the inflammatory response. By studying the levels of the inhibitory protein IκB-α, we showed that the extract and some of its constituents successfully prevented the activation of the NF-κB pathway. Several cellular events, some of them caused by LPS challenge, may trigger the activation of this pathway and among these, ROS and ER-stress are particularly relevant. As we referred above, prevention of ROS generation by the extract and its components can contribute, at least in part, to the anti-inflammatory activity found.

In order to evaluate other possible causative agent of NF-κB activation, we studied ER-stress status by evaluating the expression of the endpoint protein of the CHOP, an ER-stress marker that is known to be involved in the pathogenesis of inflammation [21], although the details and sequence of events are not completely understood [13], [22]. At this point it is important to highlight that the contribution of CHOP to the fate of RAW 264.7 cells is largely influenced by the type of pro-inflammatory *stimuli* used. When LPS is used alone, CHOP-mediated ER stress alters several metabolic and biochemical traits, though no apoptosis is detected. Differently, when LPS and IFN-γ are used in combination, NO-induced apoptosis takes place. Further studies showed that these differences are a consequence of a delay in the induction of CHOP by LPS, which takes place only after the activation of ER-protective factors, such as BiP, p58IPK, EDEM, derlin-1 and derlin-2 and, for this reason, an incomplete activation of branches of ER-stress involving CHOP takes place and does not lead to apoptosis. Full activation of ER-stress pathway *via* ER-stress inducers, such as thapsigargin or tunicamycin, is known to induce apoptosis in RAW 264.7 cells [23]. We demonstrated that CHOP expression levels were increased following exposure to LPS, an effect partly reverted by the extract and its components. Thus, the attenuation of ER stress that we describe herein for several compounds can contribute, at least in part, to the inhibition of the NF-κB pathway. In addition, due to the established contribution of ROS to further propagate the inflammatory response, namely NF-κB activation and subsequent expression of some inflammatory markers, the above-mentioned capacity of the extract and some of its compounds to attenuated oxidative stress may contribute to the anti-inflammatory activity found.

In summary, in this work we addressed the anti-inflammatory activity of a purified extract and individual compounds present in the spiny sea-star *M. glacialis*. Overall, the extract reversed LPS-induced loss of viability in RAW 264.7 cells and increased of ROS and NO levels, as well as the expression of COX-2, iNOS and the ER-stress marker protein CHOP. A proposed mechanism for the anti-inflammatory activity found can be seen in Fig. 6.

A combination of the unsaturated fatty acids *cis*-11-eicosenoic and *cis*-11,14 eicosadienoic acids and the sterol ergosta-7,22-dien-3-ol was able to mimic the anti-inflammatory effect displayed by the extract, although different compounds were active against distinct inflammatory parameters. For example, ergosta-7,22-dien-3-ol was effective against iNOS, CHOP and IKB-α expression, while its importance for the reduction of COX-2 or ROS was limited or null, respectively. Therefore, we established the importance of the potential combined action between different classes of metabolites in order to mimic the protective effect of the extract. In addition, this is the first report regarding the anti-inflammatory properties of the sterol ergosta-7,22-dien-3-ol, as well as of the unsaturated fatty acids *cis*-11-eicosenoic and *cis*-11,14-eicosadienoic acids in this system, suggesting that *M glacialis* may be a good source of nutraceuticals to be used in inflammatory conditions.
